# Supplementary material for: A GPU-based caching strategy for multi-material linear elastic FEM on regular grids
Source: PLoS One. 2020 Oct 30;15(10):e0240813. doi: 10.1371/journal.pone.0240813 (PMC7598514; doi:10.1371/journal.pone.0240813)
Supplement: Attachment — Submitted filename: Referee report.pdf [file pone.0240813.s002.pdf]

PLOS ONE: Referee report on Manuscript PONE-D-20-11213

TITLE: A GPU-Based Caching Strategy for Multi-Material Linear Elastic FEM on Regular Grids

AUTHORS: Christian Schlinkmann, Michael Roland, Christian Wolff, Patrick Trampert, Philipp Slusallek, Stefan Diebels, Tim Dahmen

General comments

In this manuscript, the authors propose a GPU-based caching strategy for the efficient computation of finite element solutions to multi-material linear elastic problems. The proposed approach belongs to the class of *matrix-free FEMs*, in that the global stiffness- and mass matrices are not computed, thereby trading a bit of computational speed for a significant gain in memory occupation. This is achieved by solving for vertex-wise *local problems.* The main novelty of the particular mesh-free method proposed in this manuscript is that, on regular hexahedral meshes, the global problem is heavily self-similar, which translates in many local problems being equal to each other. Hence, the limited number of possible different local problems are pre-computed and stored in fast access memory.
The manuscript is well-written from the point of view of computation and implementation, but it is not self-contained from a mathematical standpoint as all the necessary preliminary definitions are omitted. Numerical experiments fully illustrate the findings, but the current presentation of the computational time comparison raises questions on the advantages of the proposed approach, as explained below. A list of minor issues is presented afterwards.
In conclusion, this manuscript is suitable for publication in PLOS ONE, provided the authors fully address the issues listed below.

Major issues

1. Presenting a novel numerical method for a PDE problem without even stating the PDE is not a good practice, even in an Engineeristic context where linear elasticity is taken for granted. Moreover, the local matrices mentioned in Table 1 should be defined. To a cross-disciplinary audience, concepts such as “linear elasticity” or “stiffness matrix” are too general and may do not lend themselves to a unique interpretation. The presentation of the numerical method should be as self-contained as possible.

2. Page 25, line 323. It should be made clear from the very beginning of the Results → Performance section that, while the experiments here

are carried out in single precision, Abaqus uses double precision. In this regard, Table 1 serves only to show the asymptotic behaviour of the speedup factor. By the way, does the speedup factor retain the same trend in double precision?

Minor issues

1. Page 8, lines 26-27. “The mesh cells can have arbitrary connectivity in order to express a wide range of shapes”. Not really *arbitrary* connectivity: in classical FEMS, the intersection of two elements cannot be a portion of a face, because hanging nodes are not allowed, as correctly explained later in the manuscript. The term “arbitrary” should be relaxed, perhaps by invoking the notion of face matching.

2. Page 9. Some bibliographical references on multigrid solvers should be added at the end of the first paragraph.

3. Page 10, liness 72-79. Is this the first example of a matrix-free FEM for linear elasticity problems, or is this an improvement of existing studies on the topic? This is well explained later in the State-of-the-Art section, but a flavour of the exact novelty of the work should be given as soon as possible.

4. Page 12, line 127. “...the updated displacements of their 27 direct neighbors”. The shape of elements (tetrahedral or hexahedral) being considered and even the number of space dimensions should be mentioned. Otherwise, the reader might struggle to understand why the direct neighbours are exactly 27. This is made explicit only at a later stage. Moreover, are nodes counted as “direct neighbours” of themselves?

5. Page 12, lines 129-130. “...enhancing cache efficiency by several orders of magnitude”. Cache efficiency is actually discussed in the numerical experiments, but a direct comparison with the method in [34] showing the “improvement by several orders of magnitude” appears to be missing.

6. Page 12, lines 133-134. “In most problems with a limited number of discrete materials”. Please provide bibliographic reference(s) to such problems.

7. Page 13, line 142. “Two distinct materials are represented with gray and blue”. The only visible colors in Fig. 1a) are red and blue.

8. Page 15, equation 1. This equation should be referenced in the text. Moreover, the whole paragraph above equation (1) (starting from “the mass matrix M”) is hard to keep in the short-term memory. Hence,

this discussion should be presented as a comment below equation (1), not as a preamble.

9. Page 15, lines 175-176. "Neumann boundaries are stored separately as a three-component vector". Why? What information does this vector exactly contain? Is there an instance of such a vector for each vertex belonging to a Neumann boundary? Or just one vector for each Neumann face?

10. Page 16, lines 202-205. The difference between the discrete problem and the vertex-based representation should be made more clear.

11. Page 17, Figure 2. The pseudocode text is cut. To avoid this, the pseudocode should be inserted as text instead that as a figure.

12. Page 29, lines 443-445. "While the experiments on the increasing numbers of materials are somewhat artificial, they serve to show that the caching strategy continues to be beneficial in cases where the original assumption of an ordered distribution of materials holds". maybe the authors mean "...an ordered distribution of materials *does not hold anymore*"?
